# Polycarbonate/Sulfonamide Composites with Ultralow Contents of Halogen-Free Flame Retardant and Desirable Compatibility

**DOI:** 10.3390/ma13173656

**Published:** 2020-08-19

**Authors:** Hangfeng Yang, Hangbo Yue, Xi Zhao, Minzimo Song, Jianwei Guo, Yihua Cui, Juan P. Fernández-Blázquez, De-Yi Wang

**Affiliations:** 1 School of Chemical Engineering & Light Industry, Guangdong University of Technology, Guangzhou 510006, China; timesoon@foxmail.com (H.Y.); zhaoxizel@outlook.com (X.Z.); 15521004802@163.com (M.S.); 15024028760@163.com (Y.C.); 2 Guangzhou Vocational and Technical University of Science and Technology, Guangzhou 510550, China; 3 IMDEA Materials Institute, c/Eric Kandel 2, 28906 Getafe, Madrid, Spain; juanpedro.fernandez@imdea.org (J.P.F.-B.); deyi.wang@imdea.org (D.-Y.W.)

**Keywords:** flame retardant, polycarbonate composites, sulfonamide, compatibility

## Abstract

A novel halogen-free flame retardant containing sulfonamide, 1,3,5,7-tetrakis (phenyl-4-sulfonamide) adamantane (FRSN) was synthesized and used for improving the flame retardancy of largely used polycarbonate (PC). The flame-retardant properties of the composites with incorporation of varied amounts of FRSN were analyzed by techniques including limited oxygen index, UL 94 vertical burning, and cone calorimeter tests. The new FR system with sulfur and nitrogen elements showed effective improvements in PC’s flame retardancy: the LOI value of the modified PC increased significantly, smoke emission suppressed, and UL 94 V-0 achieved. Typically, the composite with only 0.08 wt% of FRSN added (an ultralow content) can increase the limiting oxygen index (LOI) value to 33.7% and classified as UL 94 V-0 rating. Furthermore, the mechanical properties and SEM morphology indicated that the FRSN has very good compatibility with PC matrix, which, in turn, is beneficial to the property enhancement. Finally, the analysis of sample residues after burning tests showed that a high portion of char was formed, contributing to the PC burning protection. This synthesized flame retardant provides a new way of improving PC’s flame retardancy and its mechanical property.

## 1. Introduction

Polycarbonate (PC) is playing an important role in modern industries such as automobile industry, glass assembly, construction, electrical and electronic (E&E) by virtue of its outstanding performance including high mechanical strength, elastic coefficients, transparency and thermal stability [1,2,3,4]. Although PC has already demonstrated good flame retardant performance such as 25% limiting oxygen index (LOI) and V-2 rating for burning test (UL 94), it is still difficult to meet more stringent needs, especially in fast-growing vehicle and E&E industries [5,6,7].

Numerous flame retardants (FRs) have been developed for PC, mainly including bromine-based [8,9], nitrogen-based [10], phosphorous-based [10,11,12,13], silicon-based [11,14,15] and sulfur-based [16,17] as well as various compound elements [18,19]. Although the phosphorous-based FRs have achieved great success for enhancing PC’s flame retardancy, they are commercially disfavored. A large amount of the FRs (beyond 10% [20]) is required to achieve the optimum efficiency. Additionally, nitrogen-based and silicon-based FRs are also not efficient enough to impart V-0 rating to PC in the UL 94 test unless a relatively large amount was added. High content of FRs in PC matrix is clearly a problem for further processing of the resulting composite materials, and for efficient improvements of their flame retardancy. Sulfur-based FRs, on the other hand, were very effective in improving PC’s flame retardancy performance with low concentration. For instance, with the addition of 0.25 wt% potassium diphenyl-sulfone-3-sulfonate (KSS), the LOI value was as high as 37.0% and V-0 rating for UL 94 burning test can be achieved [21].

Mechanical properties of PC composites after incorporating FRs usually decrease [22,23], which is often due to a bad compatibility or poor interfacial interactions between PC and FRs [24]. Previously, both 1,3,5,7-tetrakis (phenyl-4-sodium sulfonate) adamantane (AS_4_) [25] and 1,3,5,7-tetrakis (phenyl-4-sulfonyl-melamine) adamantane (ASN) [26] were demonstrated to show excellent flame retardancy, and their corresponding PC composites achieved V-0 rating after the addition of 0.08 wt% AS_4_ or 0.10 wt% ASN. However, we found the notch impact strength notably decreased, compared to pure PC, and observed FRs agglomeration, indicating their poor dispersion in matrix. It was deduced that the ionic groups in FRs were counterproductive, which is difficult to achieve a better FRs dispersion in polymer matrix.

The objectives of this work are three-folds: firstly, we aimed to design and produce a new halogen-free flame retardant used for effectively improving the flame retardancy performance of PC, and the improvement can be achieved just with a tiny amount of FR addition; secondly, improved mechanical properties of the resulting PC composite with the same FR incorporation are also expected; thirdly, good FR dispersion in PC matrix without agglomeration and thus desirable compatibility between PC and FR would be accomplished. To this end, a sulfur and nitrogen based FR, 1,3,5,7-tetrakis (phenyl-4-sulfonamide) adamantane (FRSN) centered on adamantane (owing to its ultrahigh physicochemical stability [27,28]) was synthesized. Hydrogen bonds potentially formed between PC and FRSN contribute to improving their interfacial bonding and compatibility. The chemical structure, flame retardant performance and notch impact strength of the PC/FRSN composites were characterized and measured. Effective improvements on these properties were found when an ultralow content (only 0.08 wt%) of this halogen-free FRSN was added into PC matrix.

## 2. Materials and Methods

### 2.1. Materials

Polycarbonate pellet 301-10 purchased from Dow Chemical Company (Midland, MI, USA) was air-dried at 120 °C for at least 8 h before uses. KSS provided by Guangdong Ever Sun Corp. (Dongguan, China) was used as a reference FR. Chlorosulfonic acid (99%) and ammonia solution (25%, AR), and 1-bromoadamantane were used as received from Energy Chemical Co. (Shanghai, China), and Macklin Biochemical Co., Ltd. (Shanghai, China), respectively. 1,3,5,7-tetraphenyladamantane was synthesized according to our previously reported procedures [29,30].

### 2.2. Methods

**Scheme 1:**
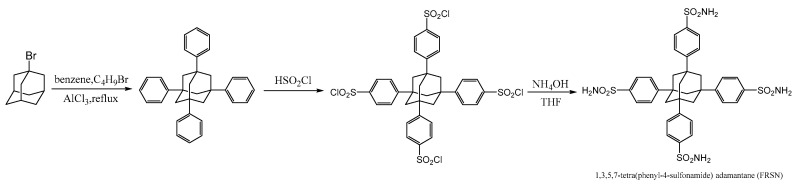
Synthetic routes and structure of the FRSN.

The FRSN was successfully synthesized from 1,3,5,7-tetraphenyladamantane through a two-step reaction, as shown in Scheme 1.

#### 2.2.1. Synthesis of 1,3,5,7-tetrakis (phenyl-4-sulfonyl chloride) adamantane

In a 100 mL three-neck flask, 1.63 g 1,3,5,7-tetraphenyladamantane (3.70 mmol) and 25 mL dry dichloromethane were added, and the flask was equipped with a dropping funnel, a tail gas unit and a reflux condenser consisting of calcium chloride drying tube on the top. The flask was placed in an ice bath before 2.5 mL chlorosulfonic acid (37.63 mmol) was added in drops. After stirring for 10 min, the mixture as stirred for 75 min after the temperature was adjusted to reach 35 °C. Once cooling down to room temperature, a light-yellow powder was obtained by centrifuge and rotary evaporation, which was then washed by water. After filtration and vacuum-dried at 45 °C for 12 h, 1,3,5,7-tetrakis (phenyl-4-sulfonyl chloride) adamantane (2.75 g, 89.0% yield) was obtained.

#### 2.2.2. Synthesis of 1,3,5,7-tetrakis (phenyl-4-sulfonamide) adamantane (FRSN)

25 mL ammonia solution was placed in the 100-mL three-neck flask placed in an ice bath. 1.1 g (1.32 mmol) 1,3,5,7-tetrakis (phenyl-4-sulfonyl chloride) adamantane dissolved in 25 mL tetrahydrofuran was added dropwise at room temperature. The aqueous layer was obtained with separatory funnel after stirring the mixture for 16 h. By removing water through rotary evaporation and vacuum drying, the final product FRSN, a light-yellow powder, was obtained (0.48 g, 48.1% yield).

#### 2.2.3. Preparation of PC/FRSN Composites

Firstly, PC and FRSN were dried overnight in a vacuum oven at 110 °C and 60 °C, respectively. PC and 0.06 wt%–0.12 wt% FRSN were mixed in a HAAKE PolyLab System (HAAKE, Thermo Fisher Scientific, Waltham, MA, USA), including PolyLab QC system, Rheomix 3000 mixer and Rheomex CTW100 QC compounder and feeding system under 50 rpm at 250 °C for 10 min. Hot press molding (15 MPa, 250 °C) was followed, and the size of composites was designed by various test standards (127 × 12.7 × 3 mm^3^ for UL 94 tests, 100 × 6.5 × 3 mm^3^ for LOI tests, 100 × 100 × 3 mm^3^ for cone calorimetry tests, and 80 × 10 × 4 mm^3^ with A type notches for notch Izod impact tests). Samples incorporating varied FRSN contents were denoted as PC/FRSN (0.06 wt%), PC/FRSN (0.08 wt%), PC/FRSN (0.1 wt%), PC/FRSN (0.12 wt%) and PC/FRSN (0.14 wt%). Pure PC and PC mixed with 0.08 wt% KSS were prepared at same conditions and denoted as PC, PC/KSS (0.08 wt%).

#### 2.2.4. Characterization and Measurement

^1^H-NMR experiments were conducted on a Bruker AVANCE III 400 MHz superconducting spectrometer (Bruker Biospin AG, Fallanden, Switzerland) using dimethylsulfoxide-d_6_ (DMSO-d_6_) as a standard solvent. The FTIR spectra were obtained from a Thermo Electron Nicolet-6700 spectrometer (Thermo Nicolet Corporation, Waltham, MA, USA) after 16 scans at 4 cm^−1^ spectrum resolution.

UL 94 vertical burning tests following the ASTM D3801-10 standard were performed on AG5100A vertical-horizontal burning apparatus (Zhuhai Angui Test Equipment Co., Ltd., Zhuhai, China).

The LOI tests in accordance with the ASTM D2863-010 standard were carried out on a HP6115 type oxygen index apparatus with a magneto-dynamic oxygen analyzer (Foshan, China).

The cone calorimeter test (CCT) evaluating flammability of polymeric materials ([31,32], was performed by a cone calorimeter, Fire Testing Technology, Ltd. (East Grinstead, UK) following the ISO5660 standard. Each specimen was exposed horizontally to an external heat flux of 35 kW·m^−2^ within aluminum foil. The values of time to ignition (TTI, s), heat release rate (HRR, kW·m^−2^), total heat release (THR, MJ·m^−2^) specific extinction area (SEA, m^2^·kg^−1^), total smoke release (TSP, m^2^), and CO production rate (COP, g·s^−1^) were recorded, and analyzed in the main text (Section 3.2.2).

The notched Izod impact strength was tested according to Chinese GB/T 1843-2008 standard by CJU 22 type notch impact testing machine (Chengde, China). The retention width at the gap is 8 mm.

SEM experiments were performed on a high resolution field-emission scanning electron microscope, Hitachi SU8010 (Tokyo, Japan). Sample surfaces were coated with a thin layer of gold prior to collecting SEM images from samples after the UL 94 burning and notch impact measurements.

Thermogravimetric analysis (TGA) experiments were conducted on a STA409PC thermal analyzer, NETZSCH Group (Selb, Germany). TGA data were collected when samples were heated from room temperature to 800 °C with a heating of 10 °C·min^−1^ under air atmosphere.

## 3. Results and Discussions

### 3.1. Chemical Structure of FRSN

**Figure 1:**
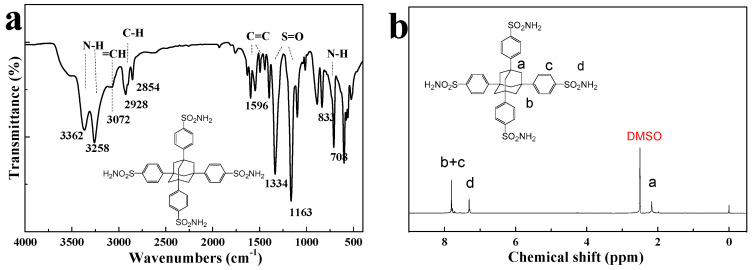
(**a**) FTIR spectrum and (**b**) ^1^H NMR spectrum of FRSN.

FTIR and ^1^H-NMR spectra of FRSN were displayed in Figure 1. As shown in Figure 1a, the stretching vibrations of = C–H and C = C in benzene rings appeared at 3072, 1596 and 1494 cm^−1^, respectively. Peaks at 833 cm^−1^, and 2928 cm^−1^ and 2854 cm^−1^ were ascribed to the characteristic absorption peak of para-disubstituted benzene, and −CH_2_ of adamantine, respectively [33]. Peaks at 1334 cm^−1^ and 1163 cm^−1^ were assigned to the stretching vibrations of S=O from the sulfamide group [34]; and peaks at 3362 cm^−1^ and 3258 cm^−1^ corresponded to the stretching vibration of N-H [35], which also had a deformation vibration peak at 708 cm^−1^.

Figure 1b shows ^1^H NMR spectrum of the flame retardant FRSN. Chemical shift peak at 2.17 ppm (a, 12H) were attributed to H proton of -CH_2_- in adamantane. The peaks at 7.75—7.85 ppm (b + c, 16H) were ascribed to the protons on benzene ring, and the chemical shift of -NH_2_ was observed at 7.31 ppm(d, 8H) [36,37,38]. Both the FTIR and NMR spectra suggested that the t FRSN flame retardant was successfully synthesized as designed.

### 3.2. Combustion Tests of the PC/FRSN Composites

#### 3.2.1. LOI and UL 94 Test

LOI and UL 94 vertical burning level of the PC/FRSN composites are shown in Table 1. It could be seen that the LOI values of the composites increased from 25.8% to 28.1% when 0.06 wt% FRSN was added. The LOI value showed an increase between pure PC and PC/FRSN (0.08 wt%). The best dosage of FRSN was 0.08 wt%, where LOI value achieved to its maximum (33.7%). Although LOI value did not progressively increase afterwards, it was still higher than 30% with the addition of FRSN contents ranging from 0.08 to 0.12 wt%. Higher addition of FR can lead to severe agglomeration within PC matrix, which would worsen the PC/FRSN adhesion or compatibility, and thus influencing the composite’s flame retardancy performance; as a consequence the LOI value decreases when FR content is higher than 0.08 wt%. This agglomeration also showed an important effect in mechanical properties as it is discussed in Section 3.5.

Such flame retardants are extremely hard to ignite according to JIS K7201 industrial standard. As for the vertical burning test, the UL 94 rating of pure PC lies in the V-2 level. However, as the addition content of FRSN ranged from 0.08 wt% to 0.12 wt%, all composites incorporating FRSN content varied from 0.08 wt% to 0.12 wt% can reach V-0 level of the UL 94 rating standard. Again, the optimum amount of FR addition was 0.08 wt%. As a comparison, adding the same amount of KSS (0.08 wt%) into PC, the PC/KSS composite only passed UL 94 V-2 rating (against V–O rating) and the LOI value only increased to 27.1% (against 33.7%).

#### 3.2.2. Cone Calorimeter Test

The cone calorimeter test results were summarized in Table 2. Specifically, the TTI value of PC/FRSN composite was slightly lower than that of pure PC, indicating a weaker thermal stability compared to PC. The sulfonate flame retardant normally promotes the decomposition of PC [39], therefore shortening the time to ignition.

**Figure 2:**
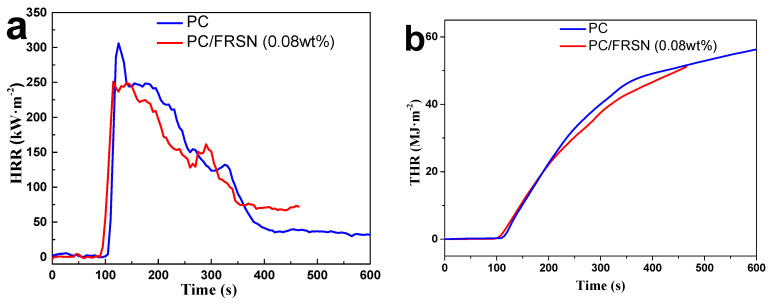

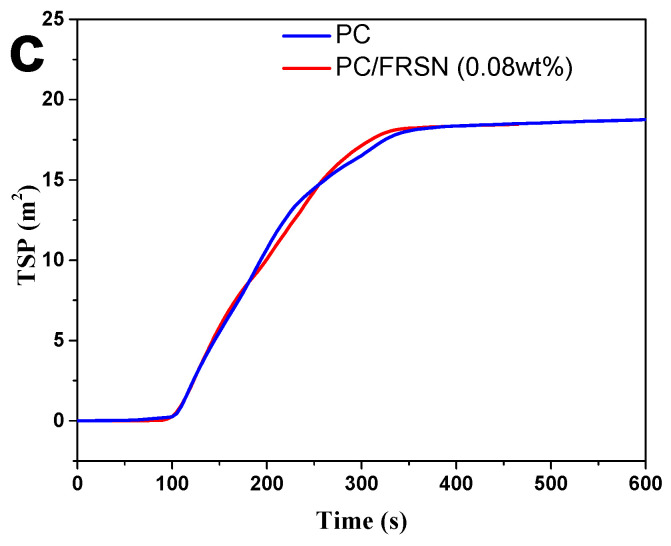
(**a**) HRR, (**b**) THR, and (**c**) TSP curves of PC and PC/FRSN (0.08 wt%).

HRR, in particular the peak of HRR (pk-HRR) value, was applied to evaluate fire safety of samples. Figure 2a shows that the addition of FRSN inhibit the combustion of the composite, i.e., lower pk-HRR (251 kW·m^−2^) versus 313 kW·m^−2^ (pure PC), reduced by 20%. Different from the triangular and single peak of HRR curve at the time range of 100-400 s for pure PC, the HRR curves of PC/FRSN (0.08 wt%) descended more slowly, indicating the lower intensity and stable combustion state. In addition, the THR value for pure PC is 61 MJ·m^−2^, whilst that for PC/FRSN (0.08 wt%) is 51 MJ·m^−2^, clearly suggesting that the total heat release is suppressed after addition the FRSN.

Smoke and toxic gas release performances in fire safety assessment [40] were also shown in Table 2. SEA of PC/FRSN (0.08 wt%) decreases to 580 m^2^·kg^−1^ against 680 m^2^·kg^−1^ for pure PC. In addition, the TSP of pure PC was slightly higher in absence of FRSN. The lower values of SEA and TSP revealed that the incorporation of FRSN might retrain the smoke emission of PC composites. Overall, the cone calorimetry analysis suggests that both heat and smoke release of PC combustion could be reduced by adding only 0.08 wt% FRSN.

### 3.3. Thermogravimetric Analyses (TGA)

**Figure 3:**
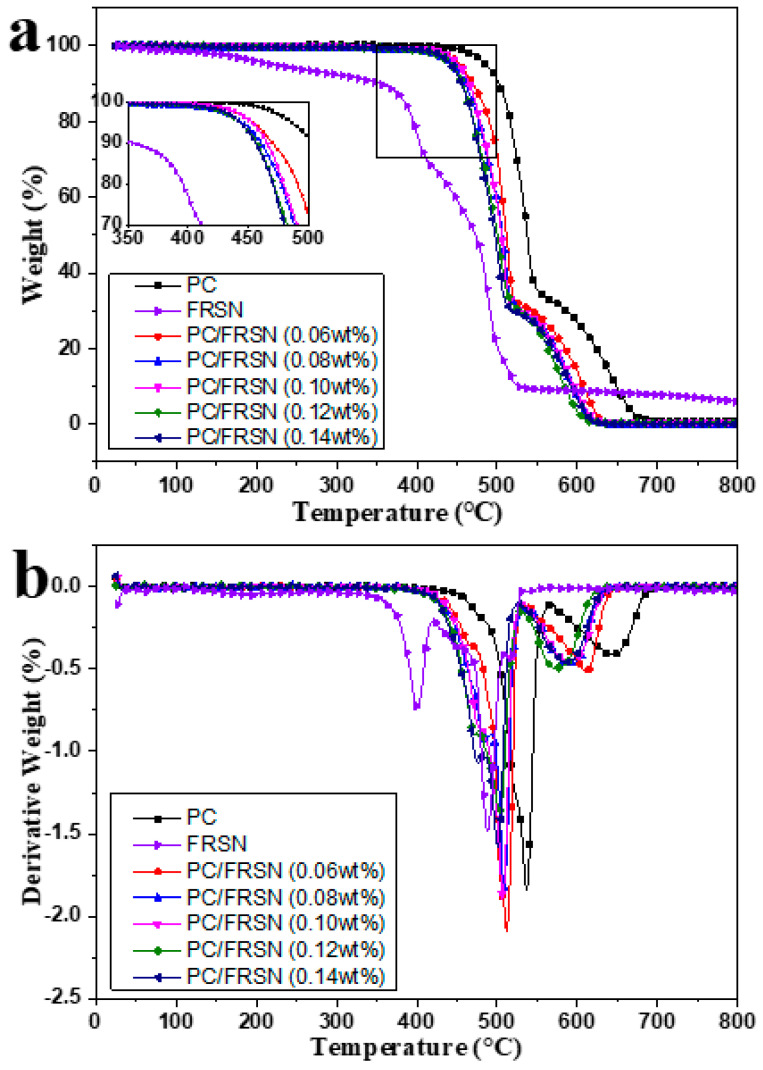
(**a**) TG and (**b**) DTG curves of PC composites, neat PC and FRSN under air atmosphere.

TGA results under air atmosphere were plotted in Figure 3, and details of thermal stability parameters such as initial decomposition temperature (T_5%_), temperature at first and second maximum mass loss rate (T_max1_, T_max2_) and char residues at 800 °C were listed in Table 3.

As shown in Figure 3, the FRSN went through a three-step decomposition process under air. In the first step, its TG curve declined slowly from 25 °C to 350 °C, with less than 10% weight loss resulting from the evaporation of water/moisture absorbed by FRSN. Then it fell sharply till about 525 °C, after which it decreased mildly. The weight loss dropped sharply in the second part was supposed to be the decomposition of sulfonamide groups on benzene rings. On this stage, there were also two decomposition peaks on the differential thermogravimetric (DTG) curve, indicating that amide group decomposes and releases nitrogen gas. The latter and broader one were due to the decomposition of the rest, producing SO_2_. The residue would be the char of benzene rings and adamantane. Usually, the decomposition temperature of additive flame retardants must not decompose at processing temperature of their correspondent engineering plastics. The decomposition temperature of the synthesized FRSN in this study is 375 °C, 125 °C higher than PC’s processing temperature (around 250 °C).

It is noted that T_5%_ and T_max1_ temperatures of PC/FRSN composites were lower than that of pure PC. The more FRSN added to PC, the lower T_max1_ of the resulting composite, suggesting the FRSN’s effect on the PC decomposition. In the oxidation process of sulfonate groups, an acidic compound SO_2_ is commonly released, which frequently has an impact on the thermal decomposition of PC by promoting the char forming process of PC [41,42]. It is also noted that the thermal stability of samples was investigated by TGA under air atmosphere, which resembles real combustion process in an open air environment and thus can evaluate the effectiveness of the flame retardant for PC matrix. Nevertheless, it is worth known that polymers go through anaerobic pyrolysis upon heating in an inert atmosphere (like nitrogen) without involving oxidative decomposition, leading to slower decomposition and better stability than that in air atmosphere.

### 3.4. Analysis of Residual Char

**Figure 4:**
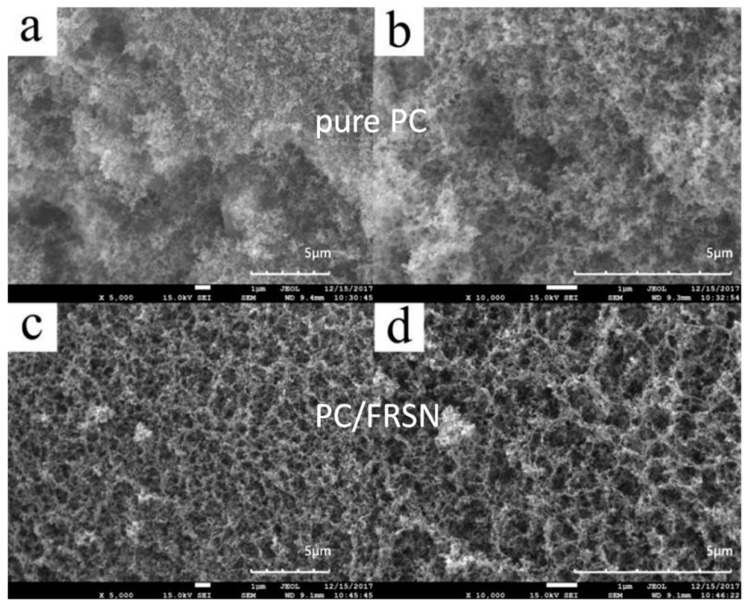
SEM micrographs at different magnifications of the residual char of (**a**,**b**) PC and (**c**,**d**) PC/FRSN after UL 94 vertical burning test.

The morphology of residual char obtained from pure PC and PC/FRSN (0.08 wt%), after their UL 94 vertical burning tests was further analyzed by SEM to better understand the char forming process during combustion. Comparing SEM images of PC and the composite, shown in Figure 4a–d, respectively, one can find clear differences: the PC char layer after burning is thinner and possesses smaller (submicron) pores, while the char layer of PC/FRSN (0.08 wt%) is thicker and forms larger (close to 1 μm) pores giving rise to a porous network carbon structure. The protective char layer with the porous and expanded carbon structure in the composite can effectively isolate burning heat from outside, and prevent air from penetrating into and reaching PC matrix during combustion. As a result, the PC/FRSN (0.08 wt%) composite would show improved flame retardancy performance and better thermal stability compared to neat PC, which is in line with experimental combustion and TGA results.

**Figure 5:**
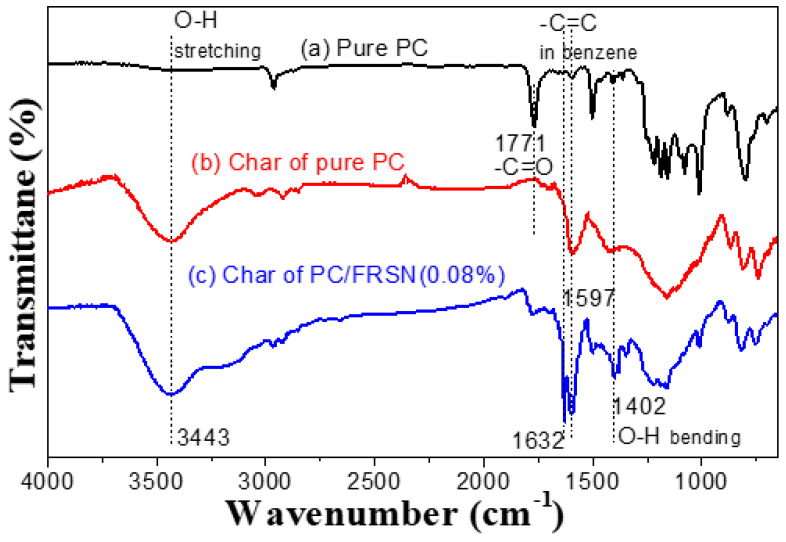
FTIR spectra of (**a**) pure PC, (**b**) residue char of pure PC and (**c**) residue char of PC/FRSN (0.08 wt%) (**c**).

The residue char of PC and PC/FRSN specimen was further analyzed by FTIR, as shown in Figure 5. Interestingly, the spectra from PC and PC/FRSN char are almost identical, but they are largely different from that of pure PC. The absorption peaks at 3443 cm^−1^ and 1402 cm^−1^ represent the stretching and plane bending vibration of -OH groups, respectively. Peaks at 1632 cm^−1^ and 1597 cm^−1^ are attributed to the −C=C− in benzene rings, which indicates that some aromatic alcohol compounds might be produced during combustion. Furthermore, pure PC lost all carbonyl groups after combustion for the reason that the C=O absorption peak at 1771 cm^−1^ in (b) in Figure 5 vanished.

### 3.5. Mechanical Properties and Dispersity of FRSN in tPC matrix

**Figure 6:**
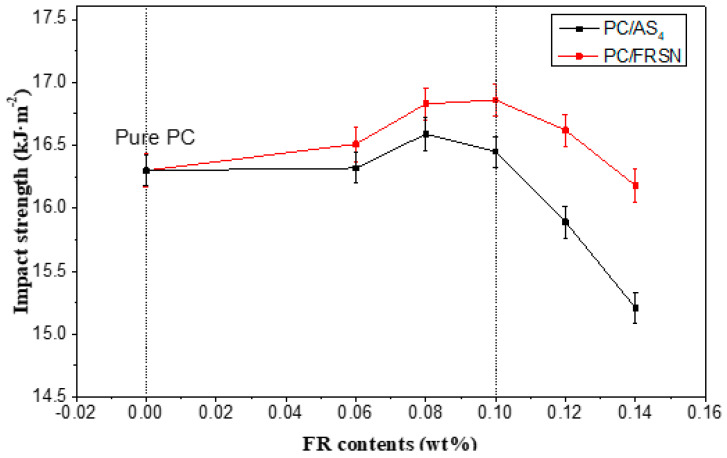
Notch impact strength of PC composites with different FR contents.

Standard notch impact tests were carried out, and the impact strength was evaluated as a function of FRSN or AS_4_ content, using PC/AS_4_ system [25] for comparison. Figure 6 shows that tendency of the impact strength of PC/AS_4_ composites with increasing the FR content [25], is quite similar to that of PC/FRSN composites (red line). Additionally, the strength is slightly enhanced after FR addition in low adding contents, compared to pure PC (16.3 kJ·m^−2^), and the PC/FRSN composite incorporating low FR content exhibited an overall higher notch impact strength. Higher addition of FR, however, may lead to severe agglomeration within matrix, which would weaken the interfacial adhesion or compatibility; thus the impact strength starts to drop when FR content is higher than 0.1 wt%. The maximum impact strength of PC/FRSN (0.10 wt%) is 16.8 kJ·m^−2^, while that for PC/AS_4_ (0.08 wt%) is around 16.3 kJ·m^−2^, indicating that the covalent groups in FRSN have better compatibility with PC than the ionic compounds in AS_4_ system.

**Figure 7:**
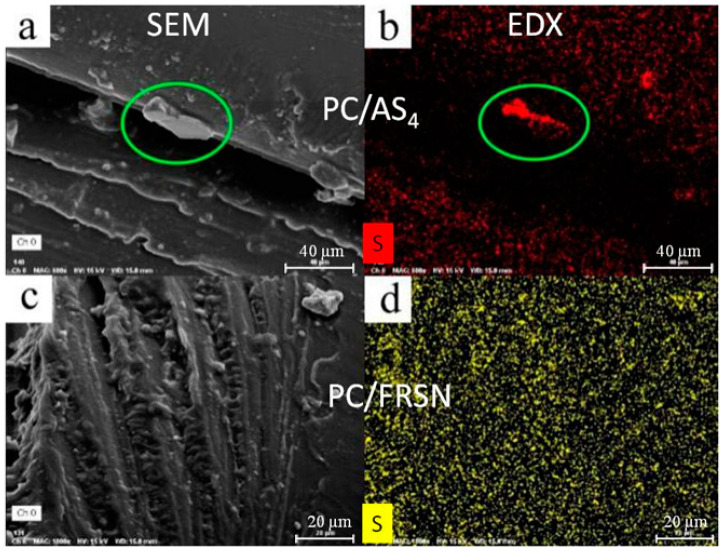
(**a**,**c**) SEM micrographs and (**b**,**d**) EDX mapping pictures of PC/AS_4_ (0.08 wt%) and PC/FRSN (0.08 wt%), respectively. Sulfur element in EDX picture is marked in red and yellow for PC/AS_4_, and PC/FRSN composite, respectively.

Cross section after the notch impact tests was observed by SEM, along with energy dispersive X-ray (EDX) mapping results. Two composite samples were selected and analyzed, PC/AS_4_ (0.08 wt%) and PC/FRSN (0.08 wt%). SEM micrographs and EDX mapping picture of the sulfur element at the same location are shown in Figure 7. There were obvious granules at the center of Figure 7a, while red dots are observed at the same place in corresponding EDX mapping image, see Figure 7b. Therefore, flame retardant AS_4_ would agglomerate even at an ultralow addition (0.08 wt%). On the contrary, only small granules can be found in Figure 7c with no concentrated sulfur element at the same location of its EDX mapping picture, see Figure 7d.

**Figure 8:**
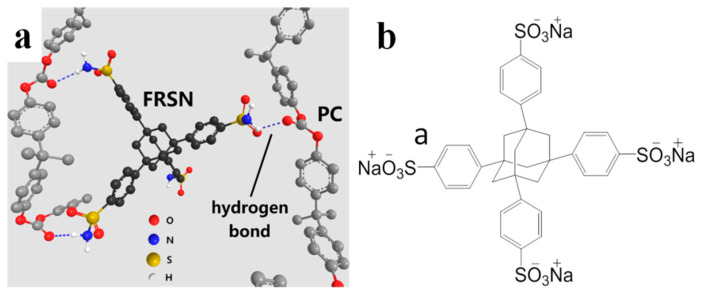
(**a**) A Schematic of hydrogen bonds formed between the flame retardant FRSN and PC, and (**b**) structure of flame retardant AS_4_.

To better explain the differences in mechanical properties between the PC/FRSN and PC/AS_4_ composites, attention has been paid to details of their structural analysis. Firstly, it is evident that the well distributed FRSN within PC matrix (see Figure 7d) imparts good PC/FRSN compatibility and interfaces of the composites, in comparison to PC/AS_4_ system. It is expected that good dispersion of FRSN in PC matrix enhances the composite’s mechanical properties (notch impact strength in our case, as well as tensile strength [23]). The notch impact strength of PC/FRSN composites was always better than that of PC/AS_4_ at varied FR contents, as shown in Figure 6. Second, we speculate that hydrogen bonds most likely formed by −NH_2_ in FRSN’s function group and–O in PC’s ester group (Figure 8a) could also play a role in enhancing the bonding interactions between PC matrix and flame retardant FRSN. On the contrary, as one can see from the structure of flame retardant AS_4_ (Figure 8b), it is of ionic bonding type, thus cannot form hydrogen bonds with PC matrix. Consequently, better interfacial bonding strength in PC/FRSN composite translates into higher mechanical (notch impact strength) properties, in comparison to PC/AS_4_ composite.

## 4. Conclusions

A novel flame retardant with sulfamide centered on adamantane (FRSN) was designed, successfully synthesized and characterized by ^1^H NMR and FTIR spectra. It was found that the FRSN with an ultralow content addition can dramatically improve the flame retardancy of polycarbonate (PC). Specifically, the LOI value of PC/FRSN composite (0.08 wt%) increased from 25.8% to 33.7%, and UL 94 V–0 rating standard was achieved. Cone calorimetry results showed that the heat and smoke release of PC were reduced with the aid of FRSN, suppressing the process of combustion and toxin emission. The decomposition of PC was promoted by SO_2_ produced during the oxidation of FRSN, forming protective char layers. With their protective char and favorable dispersion in matrix, this halogen-free flame retardant provides a novel solution to PC for enhancing its flame retardancy and mechanical properties.

## Figures and Tables

**Table 1:** Results of the LOI and UL 94 burning tests for PC and PC/FRSN composites.

Sample	LOI/%	UL 94
PC	25.8	V−2
PC/FRSN (0.06 wt%)	28.1	V−1
PC/FRSN (0.08 wt%)	33.7	V−0
PC/FRSN (0.10 wt%)	32.3	V−0
PC/FRSN (0.12 wt%)	30.8	V−0
PC/FRSN (0.14 wt%)	29.3	V−1
PC/KSS (0.08 wt%)	27.1	V−2

**Table 2:** Cone calorimetric data with heat flux of 35 kW·m^−2.^

Sample	TTI/s	pk–HRR (kW·m−2)	THR /MJ·m^−2^	SEA/ m^2^·kg^−1^	TSP/m^2^	Mean COY /kg·kg^−1^	Mean CO_2_Y /kg·kg^−1^
PC	102	313	61	680	19	0.148	1.652
PC/FRSN (0.08 wt%)	86	251	51	580	18	0.096	1.781

**Table 3:** TGA results of PC composites, neat PC and FRSN under air atmosphere.

Sample	T_5%_/°C	T_max1_/°C	Residue/wt%
PCs	485	537	0.8
FRSN	222	399	4.6
PC/FRSN (0.06 wt%)	454	512	0.1
PC/FRSN (0.08 wt%)	444	508	0.3
PC/FRSN (0.10 wt%)	452	506	0.3
PC/FRSN (0.12 wt%)	443	503	0.3
PC/FRSN (0.14 wt%)	443	501	0.1
